# Agreement between self-, mother and father proxy-reports on health-related quality of life in adolescents with Tourette syndrome

**DOI:** 10.1007/s00787-024-02418-6

**Published:** 2024-04-13

**Authors:** Isabelle Jalenques, Candy Guiguet-Auclair, Dominique Morand, Fabien Bourlot, Sophie Lauron, Nathan Mitelman, Andreas Hartmann, Fabien Rondepierre, The Syndrome de Gilles de La Tourette Study Group, C. Angonin, F. Bourlot, E. Deniau, P. Derost, L. Gerbaud, C. Guiguet-Auclair, A. Hartmann, I. Jalenques, S. Lauron, G. Legrand, A. Macleod, M. Marcheix, N. Mitelman, D. Morand, J. Müllner, C. Ramanoel, F. Rondepierre

**Affiliations:** 1 Université Clermont Auvergne, Clermont Auvergne INP, CHU Clermont-Ferrand, Service de Psychiatrie de l’Adulte A et Psychologie Médicale, Centre de Compétences Gilles de la Tourette, CNRS, Institut Pascal, F-63000 Clermont-Ferrand Cedex, France; 2 Université Clermont Auvergne, Clermont Auvergne INP, CHU Clermont-Ferrand, CNRS, Institut Pascal, F-63000 Clermont-Ferrand, France; 3 Direction de la Recherche Clinique et de l’Innovation, CHU Clermont-Ferrand, 63003 Clermont-Ferrand, France; 4 Université Clermont Auvergne, Clermont Auvergne INP, CHU Clermont-Ferrand, Service de Psychiatrie de l’Adulte A Et Psychologie Médicale, CNRS, Institut Pascal, F-63000 Clermont-Ferrand, France; 5 Département de Neurologie, Pôle des Maladies du Système Nerveux, Groupe Hospitalier Pitié-Salpêtrière, Centre de Référence ‘Syndrome Gilles de la Tourette’, 75013 Paris, France; 6 Service de Psychiatrie de l’Adulte A et Psychologie Médicale, Centre de Compétences Gilles de la Tourette, CHU Clermont-Ferrand, 63003 Clermont-Ferrand, France

**Keywords:** Gilles de la Tourette syndrome, Health-related quality of life, Adolescent self-reports, Parent-proxy-reports, Agreement

## Abstract

This study aimed to investigate agreement and discrepancies between parent proxy- and adolescent self-reports on assessments of adolescents’ health-related quality of life (HRQoL), and the role that individual factors may play in parent-adolescent agreement, in a sample of adolescents with Tourette syndrome (TS) compared to a control group of healthy adolescents. Adolescents aged 12–18 years diagnosed with TS were recruited with their parents from primary and secondary referral centres. Adolescent healthy controls were matched for gender and age. Adolescents and each of their parents completed a set of questionnaires including a HRQoL evaluation of adolescent, the ‘Vécu et Santé Perçue de l’Adolescent’. Mother-adolescent, father-adolescent and mother-father agreements on adolescents’ HRQoL scores were investigated at individual and group level, both in TS and control groups. Data were available for 75 adolescents, 75 mothers and 63 fathers, in the TS group. Agreement between mother, father proxy-reports and TS adolescents self-reports of HRQoL varied from poor to good, without significant difference with the control group. In TS group, mothers and fathers underestimated adolescents’ HRQoL in ‘Psychological well-being’ subscale and mothers underestimated it in ‘Physical ‘well-being’ subscale, while controls overestimated adolescents’ HRQoL in these subscales. Larger mother-adolescent discrepancies for ‘Psychological well-being’ and ‘Physical well-being’ subscales were associated with internalizing symptoms. Regarding future studies, comprehensive evaluation of the various dimensions of adolescents’ HRQoL with TS requires the integration of the perspectives of both adolescents, mothers and fathers. Clinicians should take into account this point to provide comprehensive care and services.

**Supplementary Information:**

The online version contains supplementary material available at 10.1007/s00787-024-02418-6.

## Introduction

Gilles de la Tourette syndrome (TS) is defined by the DSM-5 as a chronic neuropsychiatric disorder, characterized by multiple motor and one or more vocal tics, having started under the age of 18 years and persisting for more than 1 year since the first tic onset, after excluding secondary cause [1]. TS prevalence has been estimated between 0.3 and 0.7% in school-aged children [2, 3]. Comorbid conditions (attention-deficit and hyperactivity disorder (ADHD), obsessive–compulsive disorder (OCD) notably) are associated in around 90% of children [4]. Tics and comorbid conditions can affect health-related quality of life (HRQoL) in youth with TS and chronic tic disorder [5].

Several systematic reviews of the literature point out that the levels of agreement in HRQoL assessments between children in various clinical sample and their parents may vary and that this needs to be addressed when assessing the child’s HRQoL and planning interventions [6–9].

Among HRQoL studies in youth with TS and chronic tic disorder, scarce ones have compared self and parent ratings of children HRQoL and their results seem to diverge. Two studies showed good agreement between the self-assessment of children’s HRQoL and the parents-proxy assessment for all dimensions, despite differences in HRQoL scores between the two assessments [10, 11]. On the contrary, two other studies showed no correlation [12, 13]. However, in one of these studies, children and parents did not complete the same questionnaire [12]. Finally a study found for children aged 8–11 years, strong positive correlations between parents and child ratings on each HRQoL domain whereas for children aged 12–17 years, no significant correlations were found [5].

The differences between the results of these studies could be partly explained by sociodemographic and methodological aspects: the sample size of the studies which included young children up to adolescents, without presenting in most studies differentiated results according to age; the absence of description of which parents rating the questionnaires; the lack of comparison between the responses of mothers and fathers; and differences in the statistical tests used.

Moreover, several authors compared patient results to normative data or to control data from other studies, but agreement in clinical child-parent dyads was no longer compared to agreement in a healthy control group [5, 10].

Finally, the age of the children is an important factor to take into account to evaluate the agreement between parents and children. Indeed, the adolescence is a developmental process where parent–child relationships change. The adolescents need more autonomy to build oneself personally while the parents need to adapt their behavior. During this period, differences of point of view (between parents and adolescents) may appear, which can lead to conflicts and to affect the assessment of parent-adolescents agreement [14].

Thus in the current explorative study, we investigated agreement between parents and adolescents with TS on reports of adolescents’ HRQoL, and the role that individual factors may play in parent-adolescent agreement, in a sample of adolescents with TS aged 12–18 years old and a control group of healthy adolescents and their parents. The aims of this study were: (1) to evaluate the degree of agreement on adolescents’ HRQoL scores between both the mothers and fathers proxy-reports and self-reports of adolescents with TS; (2) to assess the direction and the magnitude of discrepancies in TS adolescents’ HRQoL scores in mother-adolescent, father-adolescent and mother-father dyads; (3) to compare agreement and discrepancies in mother-adolescent, father-adolescent and mother-father dyads between TS and control groups; (4) to evaluate potential factors that might be associated with higher discrepancies in TS dyads.

## Methods

### Study design and participants

The design of this controlled study was previously described in details elsewhere [15]. Over a period of 3 years in France, before the COVID-19 pandemic, adolescents aged 12–18 years diagnosed with TS according to DSM-IV-TR criteria were recruited with their parents (TS family) from primary and secondary referral centres during a consultation. Exclusion criteria for adolescents and parents were intellectual disability according to DSM-IV-TR criteria and inability to understand or complete the questionnaires.

During this consultation, a neurologist assessed the severity of the tics of TS adolescents using the Yale Global Tic Severity Scale (YGTSS) [16] and a psychiatrist assessed their obsessive–compulsive symptoms using the Children’s Yale-Brown Obsessive–Compulsive Scale (CY-BOCS) [17].

Adolescent healthy controls without TS matched for gender and age and their parents were also recruited. The control family was chosen by the TS family to take part in the study. They had to live in the same region, be composed of the same number of children and have no family relationship with the TS family.

A set of questionnaires was mailed with a return envelope to all the adolescents and each of their parents (mothers and fathers) 2 weeks after the consultation during which they were recruited for the TS group, and 2 weeks after giving their informed consent to participate for the control family. The adolescents, mothers and fathers were instructed to self-complete the questionnaires independently.

The study was approved by the French Committee for the Protection of Individuals southeast 6 (reference CPP AU803, 30 November 2009) and was conducted in accordance with the Declaration of Helsinki. All parents gave their written informed consent. Consent for minor adolescents was obtained from their parents prior to participation.

### Measures

#### HRQoL of adolescents

The HRQoL of adolescents during the previous 4 weeks was assessed by the ‘Vécu et Santé Perçue de l’Adolescent’ questionnaire [18], which comprises 37 items grouped into ten subscales: ‘Vitality’ (5 items), ‘Psychological well-being’ (5 items), ‘Relationship with friends’ (5 items), ‘Leisure activities’ (4 items), ‘Relationship with parents’ (4 items), ‘Physical well-being’ (4 items), ‘Relationship with teachers’ (3 items), ‘School performance’ (2 items), ‘Body image’ (2 items) and ‘Relationship with medical staff’(3 items) (not studied here). Each item is rated on a 5-point Likert scale from 1 (“not at all/never”) to 5 (“very much/always”). For each subscale, a total score is calculated as the mean of the item scores of the subscale. The scoring of item response is reversed when necessary so that higher scores indicates better HRQoL. A missing score is assigned if more than one-half of the items in each subscale are missing. All scores are linearly transformed on a scale from 0 (indicating the worst HRQoL) to 100 (the highest HRQoL). Two parallel self-administered questionnaires are available with identical items: an adolescents’ self-administered version (VSP-A) and a parents’ one to assess the HRQoL of their children (VSP-P) [19]. The items of the parents’ form were reworded following this example “Was your child anxious?” instead of “Were you anxious?”.

#### Tics and obsessive–compulsive symptoms of TS adolescents

The Motor tic, Obsessions and compulsions, Vocal tic Evaluation Survey (MOVES) was self-completed by adolescents to assess severity of their tics and related sensory phenomena observed in TS [20]. It comprises 20 items measuring the past 4 weeks’ severity of five phenomena: ‘Motor tics’, ‘Vocal tics’, ‘Obsessions’, ‘Compulsions’ and ‘Associated symptoms’ (echolalia, echopraxia, coprolalia, copropraxia). For each subscale, a score is obtained by adding the scores of the items listed in the subscale. A total MOVES score is calculated by adding the scores of these five subscales, with range from 0 (no symptom) to 60 (the worst condition). The ‘Motor tics’ and ‘Vocal tics’ scores are added to form a ‘Tic’ subscale score. The ‘Obsessions’ and ‘Compulsions’ scores are added to form an ‘Obsessions- Compulsions’ subscale score.

#### Behavioural and emotional problems of adolescents

The Child Behaviour Checklist (CBCL) for ages 6–18 years was self-completed by both mothers and fathers to assess the presence of adolescents’ behavioural and emotional problems [21]. It is a useful screening-diagnostic tool to identify the main psychiatric and behavioural problems in TS [22]. The CBCL provides scores for three broad-band subscales: ‘Internalizing symptoms’, ‘Externalizing symptoms’ and ‘Total problems’. Higher scores for each subscale denotes greater problems. Raw scores were transformed into T-scores to obtain for adolescents the nonclinical, borderline clinical and clinical profiles according to standardization and cut-offs [23].

#### HRQoL of parents

Parents’ HRQoL was assessed by two self-administered questionnaires, the Medical Outcomes Study Item Short Form Health Survey (SF-36) [24] and the World Health Organization Quality of Life Brief (WHOQOL-BREF) [25] questionnaire. The SF-36 consists of 36 items assigned to eight multi-item subscales: ‘Physical functioning’, ‘Role physical’; ‘Bodily pain’, ‘Vitality’, ‘Mental health’, ‘Role emotional’, ‘Social functioning’ and ‘General health’. The WHOQOL-BREF comprises four subscales: ‘Physical health’, ‘Psychological health’, ‘Social relationships’ and ‘Environment’. For each subscale of the SF-36 and WHOQOL-BREF, scores between 0 and 100 are established, with higher values indicating better HRQoL.

#### Psychiatric morbidity of parents

Anxiety and depressive symptoms of parents were assessed by the Hospital Anxiety and Depression Scale (HADS) [26], a self-report scale consisting of 14 items, 7 related to anxiety (HADS-A) and 7 to depression (HADS-D). For both depression and anxiety, a total score ranging from 0 to 21 is calculated, with higher scores representing a higher level of symptoms of depression and anxiety. The HADS scores can also be interpreted by cut-off scores, with a score strictly higher than 7 indicating a possible or probable clinical case [27].

#### Demographic and clinical information

Sex of adolescents, age, and clinical data on TS were collected: time since first symptoms, time since diagnosis, medical treatment, follow-up care and current health problems. For each of the parents, age, marital status, level of education, family size, current health problems, medical treatment and family medical history in connection with TS were collected.

### Statistical analysis

All analyses were performed with SAS software (version 9.4, SAS Institute, Cary, NC, 2002–2012) and conducted at a two-sided alpha = 0.05 significance level. Because of the explorative nature of our study, no adjustment for multiple testing was done (this would have overestimated the role of chance) [28, 29].

Continuous variables were presented as means and standard deviations and categorical variables as numbers and percentages.

Mother-adolescent, father-adolescent and mother-father agreements on adolescents’ HRQoL scores were investigated at the individual and group level, both in the TS and control groups.

At the individual level, the intraclass correlation coefficients (ICCs) using two-way mixed effects models with absolute agreement definition were calculated per dyad. Values of ICC inferior to 0.19 were interpreted as poor agreement, between 0.20 and 0.39 as fair agreement, between 0.40 and 0.59 as moderate agreement, between 0.60 and 0.79 as good agreement, and values equal or superior to 0.80 as excellent agreement [30]. The ICCs were compared based on their 95% confidence intervals.

At the group level, different approaches were used to assess agreement in the TS group. First, mean absolute difference between scores (proxy minus adolescent scores, mother minus father scores, irrespective of the direction of the discrepancies) and mean directional difference (showing the direction of the discrepancies) were calculated. A negative mean difference shows lower parent-proxy report of HRQoL compared to adolescent self-reported scores or lower mother-proxy report compared to father proxy-report. Second, Student paired *t* tests were used to assess differences between HRQoL scores in mother-adolescent, father-adolescents and mother-father dyads. Third, effect sizes (ES) were used to evaluate the magnitude of the directional differences for paired observations, and were defined as the ratio of the mean difference to its SD. A negative ES indicated a lower level of adolescents’ HRQoL reported by parents compared to adolescents or reported by mothers compared to fathers. Effect sizes can be interpreted as: negligible for |< 0.20|, small for |0.20–0.49|, moderate for |0.50–0.79|, and large for |≥ 0.80| [31].

Directional differences in each dyad were compared between TS and control groups using unpaired Student *t* tests.

The HRQoL subscales for which parents of TS adolescents had significantly differences in discrepancies between mother-adolescent, father-adolescent and mother-father dyads in comparison to control parents were included in the analysis of demographic and clinical factors affecting concordance in dyads. Multivariate linear regression models with a forward selection were used to test the effect of individual variables on dyads agreement for each HRQoL subscale separately. We included in the model independent variables significant in the bivariate analyses at p < 0.15. Directional differences were the dependent variables. Independent variables were adolescents’ factors (gender, age, time since first symptoms and diagnosis, treatment reported for tics, VSP-A, YGTSS, CY-BOCS, MOVES, and CBCL scores) and parental factors (age, level of education, family size, health problems, SF-36, WHOQOL-BREF and HADS scores).

## Results

### Characteristics of parents and adolescents

Data were available for 75 adolescents, 75 mothers and 63 fathers, and 63 dyad mother-father of the same child in the TS group and for 75 adolescents, 75 mothers and 62 fathers, and 62 dyad mother-father in the control group. Sociodemographic and clinical characteristics of parents and TS adolescents are detailed in Table 1. The characteristics of parents and adolescents in the control group are detailed elsewhere [15].

**Table 1:** Sociodemographic and clinical characteristics of adolescents and parents with TS

	TS group	
	Mothers (n = 75)	Fathers (n = 63)
*Adolescents’ gender, n (%)*		
Male	60 (80.0)	
Female	15 (20.0)	
Adolescents’ age, mean (SD)	14.8 (1.8)	
Time since first symptoms (years), mean (SD)	8.1 (3.5)	
Time since diagnosis of TS (years), mean (SD)	4.0 (3.0)	
*Treatment reported for tics*, *n* *(%)*	54 (72.0)	
Neuroleptics/Antipsychotics	51 (68.0)	
Others	8 (10.7)	
*Other treatment reported,* *n* *(%)*	19 (25.3)	
Antidepressants	13 (17.3)	
Anxiolytics	6 (8.0)	
Attention-deficit/hyperactivity disorders	4 (5.3)	
Sleep disorders	3 (4.0)	
*Actual symptoms reported by mothers, n (%)*		
ADHD	41 (54.7)	
COD	16 (21.3)	
Learning disabilities	34 (45.3)	
Anxiety	51 (68.0)	
Depression	16 (21.3)	
Sleeping disorder	23 (30.7)	
*Medical monitoring for TS reported by mothers, n (%)*		
Psychiatrist or child psychiatrist	34 (45.3)	
Psychologist	28 (37.3)	
*YGTSS scores, mean (SD)*		
Motor tics	13.2 (4.8)	
Vocal tics	9.4 (6.1)	
Overall impairment	11.3 (13.7)	
Total	33.9 (20.0)	
*CY-BOCS scores, mean (SD)*		
Obsessions	2.4 (4.6)	
Compulsions	3.6 (5.1)	
Total	6.0 (9.0)	
*MOVES scores, mean (SD)*		
Motor tics	5.4 (2.8)	
Vocal tics	2.9 (3.0)	
Obsessions	2.1 (2.5)	
Compulsions	3.0 (2.4)	
Other associated symptoms	1.2 (2.0)	
Total	14.2 (9.3)	
*CBCL scores reported by parents*		
Total problems, mean (SD)	53.6 (25.0)	44.1 (23.1)
Borderline or clinical range, n (%)	41 (57.7)	25 (41.7)
Internalizing symptoms, mean (SD)	17.6 (8.9)	14.8 (8.9)
Borderline or clinical range, n (%)	45 (61.6)	32 (52.5)
Externalizing symptoms, mean (SD)	14.3 (9.0)	11.0 (6.9)
Borderline or clinical range, n (%)	33 (44.0)	21 (33.3)
Parents’ age (years), mean (SD)	44.1 (4.7)	47.1 (6.2)
*Parents’ marital status, n (%)*		
Single	1 (1.3)	1 (1.6)
Married/living with partner	64 (85.3)	58 (92.1)
Separated/divorced	9 (12.0)	4 (6.3)
Widowed	1 (1.3)	0
*Parents’ level of education, n (%)*		
Lower than or equal to high school	44 (58.7)	37 (58.7)
Superior to high school	31 (41.3)	25 (40.3)
Family size, mean (SD)	4.0 (0.9)	3.9 (1.2)
Health problems, *n* (%)	23 (30.7)	20 (31.7)
*Medical treatment,* *n* *(%)*	29 (39.2)	19 (30.2)
Antidepressants	10 (13.3)	5 (7.9)
Anxiolytics	6 (8.0)	1 (1.6)
Hypnotics	2 (2.7)	0
Others	19 (25.3)	14 (22.2)
*SF-36 scores, mean (SD)*		
Physical functioning	87.2 (16.6)	95.2 (6.2)
Role physical	78.1 (32.3)	82.4 (26.6)
Bodily pain	74.5 (25.3)	82.5 (17.4)
Vitality	46.2 (18.7)	61.1 (14.9)
Mental health	56.0 (19.7)	66.8 (19.1)
Role emotional	68.0 (40.7)	83.0 (30.1)
Social functioning	70.1 (24.6)	79.2 (22.2)
General health	65.7 (15.7)	70.1 (15.5)
*WHOQOL-BREF scores, mean (SD)*		
Physical health	70.5 (18.0)	79.1 (12.7)
Psychological health	60.3 (17.8)	62.9 (14.9)
Social relationships	65.4 (18.7)	60.0 (24.6)
Environment	68.2 (16.0)	69.5 (15.9)
*Anxiety*		
HADS-A score, mean (SD)	8.4 (4.1)	6.8 (3.7)
Possible or probable clinical case, n (%)	42 (56.0)	20 (31.7)
*Depression*		
HADS-D score, mean (SD)	5.7 (3.7)	4.8 (4.2)
Possible or probable clinical case, n (%)	22 (29.3)	14 (22.2)

### Strength of agreement between parents and adolescents ratings of TS adolescents’ HRQoL

Table 2 presents the agreement between the TS adolescent self-reported and the parent (mother and father) proxy-reported HRQoL, as between mother and father proxy-reported HRQoL. All ICCs were significantly different from zero except for ‘Relationship with parents’ subscale, ranging from 0.07 to 0.76 in mother-adolescent dyads, from 0.13 to 0.65 in father-adolescent dyads, and from 0.02 to 0.75 in mother-father dyads. The highest ICCs were found for ‘Leisure activities’ subscale and the lowest for ‘Relationship with parents’.

**Table 2:** Agreement on adolescents’ HRQoL scores in mother-adolescent, father-adolescent and mother-father dyads as defined by the intraclass correlation coefficient (ICC) in TS and control group

VSP-A/VSP-P scales	Mother-Adolescent		Father-Adolescent		Mother–Father	
	n^a^	ICC (95% CI)	n^a^	ICC (95% CI)	n^a^	ICC (95% CI)
*Vitality*						
TS group	75	0.57 (0.39, 0.70)***	62	0.47 (0.25, 0.64)***	62	0.64 (0.47, 0.77)***
Control group	75	0.24 (0.01, 0.44) *	62	0.21 (-0.04, 0.44)	62	0.58 (0.39, 0.72)***
*Psychological well-being*						
TS group	74	0.53 (0.21, 0.72)***	61	0.34 (0.07, 0.56)***	61	0.40 (0.17, 0.60)***
Control group	75	0.40 (0.19, 0.57)***	62	0.32 (0.09, 0.53)**	62	0.60 (0.41, 0.74)***
*Relationship with friends*						
TS group	71	0.52 (0.33, 0.67)***	61	0.49 (0.27, 0.66)***	58	0.62 (0.44, 0.76)***
Control group	75	0.45 (0.25, 0.61)***	61	0.23 (-0.00, 0.44) *	61	0.46 (0.24, 0.64)***
*Leisure activities*						
TS group	75	0.76 (0.64, 0.85)***	63	0.65 (0.49, 0.78)***	63	0.76 (0.63, 0.85)***
Control group	75	0.73 (0.59, 0.82)***	62	0.63 (0.45, 0.76)***	62	0.74 (0.61, 0.84)***
*Relationship with parents*						
TS group	75	0.07 (-0.10, 0.25)	62	0.13 (-0.11, 0.36)	62	0.02 (-0.18, 0.24)
Control group	75	0.29 (0.07, 0.49)**	62	0.35 (0.11, 0.55)***	62	0.45 (0.21, 0.64)***
*Physical well-being*						
TS group	75	0.44 (0.24–0.61)***	62	0.32 (0.08–0.53)**	62	0.63 (0.46–0.76)***
Control group	75	0.26 (0.03–0.46) *	62	0.26 (0.02–0.48) *	62	0.37 (0.13–0.57)**
*Relationship with teachers*						
TS group	66	0.65 (0.48–0.77)***	53	0.56 (0.34–0.72)***	56	0.65 (0.46–0.78)***
Control group	68	0.48 (0.27–0.64)***	60	0.34 (0.10–0.55)**	58	0.42 (0.18–0.61)**
*School performance*						
TS group	69	0.57 (0.38–0.71)***	55	0.57 (0.36–0.72)***	57	0.72 (0.57–0.82)***
Control group	73	0.61 (0.42–0.75)***	60	0.43 (0.19–0.62)***	62	0.61 (0.42–0.74)***
*Body image*						
TS group	75	0.60 (0.43–0.73)***	63	0.31 (0.07–0.52)**	63	0.50 (0.29–0.66)***
Control group	75	0.32 (0.10–0.50)**	62	0.31 (0.07–0.52)**	62	0.52 (0.31–0.68)***

ICC values interpretation: poor (0–0.19), fair (0.20–0.39), moderate (0.40–0.59), good (0.60–0.79), excellent (0.80–1)

*ICC* intraclass correlation coefficient; *95% CI* 95% confidence interval.

*p < 0.05, **p < 0.01, ***p < 0.001.

^a^n: number of complete pairs, i.e. the number of dyads without missing values.

Agreement between adolescents and mothers was good for ‘Leisure activities’, ‘Relationship with teachers’, and ‘Body image’ subscales; moderate for ‘Vitality’, ‘Psychological well-being’, ‘Relationship with friends’, ‘Physical well-being’, and ‘School performance’ subscales; and poor for ‘Relationship with parents’ subscale. Agreement between adolescents and fathers was lower with moderate ICCs for ‘Vitality’, ‘Relationship with friends’, ‘Relationship with teachers’, and ‘School performance’ subscales; good ICCs for ‘Leisure activities’, and ‘Relationship with teachers’ subscales; fair ICCs for ‘Psychological well-being’, ‘Physical well-being’, and ‘Body image’ subscales; and poor ICCs for ‘Relationship with parents’ subscale. ICCs comparing mothers and fathers were good for all dimensions, except for ‘Psychological well-being’ and ‘Body image’ subscales with moderate ICCs, and ‘Relationship with parents’ subscale with a poor ICC.

### Discrepancies between parents and adolescents ratings of TS adolescents’ HRQoL

Adolescents’ HRQoL scores reported by adolescents themselves, and by mothers and fathers in TS group are shown in Supplementary Table 1.

The approaches used to assess differences between self and proxy-reports of adolescents’ HRQoL, and between mother and father-proxy reports, in the TS group, are provided in Table 3. The mean of the absolute between mother-adolescent, father-adolescent and mother-father dyads were largest for ‘Psychological well-being’, ‘Relationship with parents’ and ‘Body image’ subscales, which indicated less agreement between dyads. The mean absolute differences ranged from 13.19 to 22.64 between mothers and adolescents, from 14.70 to 26.39 between fathers and adolescents and from 11.17 to 20.04 between mothers and fathers.

**Table 3:** Direction and magnitude of discrepancies in TS adolescents’ HRQoL scores in mother-adolescent, father-adolescent and mother-father dyads

VSP-A/VSP-P scales	Complete pairs	Absolute difference^a^	Directional difference^b^	*p* value	ES (95% CI)^c^
	n	Mean (SD)	Mean (SD)		
*Vitality*					
Mother-adolescent dyad	75	15.10 (11.93)	− 4.17 (18.86)	0.0596	− 0.22 (− 0.43, − 0.01)
Father-adolescent dyad	62	15.56 (12.42)	− 2.50 (19.85)	0.3253	− 0.13 (− 0.38, 0.13)
Mother–Father dyad	62	11.17 (11.12)	− 1.90 (15.71)	0.3460	− 0.12 (− 0.33, 0.09)
*Psychological well-being*					
Mother-adolescent dyad	74	20.02 (15.29)	− 13.77 (21.17)	< 0.0001	− 0.65 (− 0.88, − 0.42)
Father-adolescent dyad	61	22.87 (18.08)	− 15.33 (24.90)	< 0.0001	− 0.62 (− 0.91, − 0.33)
Mother–Father dyad	61	16.91 (16.89)	0.35 (23.99)	0.9101	0.02 (− 0.26, 0.29)
*Relationship with friends*					
Mother-adolescent dyad	71	17.19 (16.51)	− 5.99 (23.15)	0.0325	− 0.26 (− 0.49, − 0.03)
Father-adolescent dyad	61	17.40 (17.77)	− 5.30 (24.40)	0.0949	− 0.22 (− 0.47, 0.04)
Mother–Father dyad	58	16.13 (14.24)	− 1.76 (21.55)	0.5364	− 0.08 (− 0.30, 0.14)
*Leisure activities*					
Mother-adolescent dyad	75	13.19 (10.64)	− 4.53 (16.40)	0.0193	− 0.28 (− 0.43, − 0.12)
Father-adolescent dyad	63	16.34 (12.91)	− 2.84 (20.73)	0.2804	− 0.14 (− 0.34, 0.07)
Mother–Father dyad	63	12.33 (13.27)	− 0.96 (18.16)	0.6765	− 0.05 (− 0.23, 0.12)
*Relationship with parents*					
Mother-adolescent dyad	75	22.64 (20.99)	15.86 (26.55)	< 0.0001	0.60 (0.28, 0.92)
Father-adolescent dyad	62	20.16 (16.49)	6.25 (25.41)	0.0574	0.25 (− 0.08, 0.58)
Mother–Father dyad	62	18.41 (17.21)	10.22 (23.12)	0.0009	0.44 (0.09, 0.80)
*Physical well-being*					
Mother-adolescent dyad	75	18.00 (14.42)	− 7.17 (22.01)	0.0062	− 0.33 (− 0.57, − 0.09)
Father-adolescent dyad	62	19.25 (13.97)	− 2.52 (23.78)	0.4072	− 0.11 (− 0.40, 0.18)
Mother–Father dyad	62	12.50 (11.82)	− 3.43 (16.93)	0.1160	− 0.20 (− 0.42, 0.01)
*Relationship with teachers*					
Mother-adolescent dyad	66	13.89 (12.43)	1.77 (18.63)	0.4437	0.10 (− 0.11, 0.30)
Father-adolescent dyad	53	14.70 (13.79)	− 1.34 (20.21)	0.6323	− 0.07 (− 0.31, 0.18)
Mother–Father dyad	56	13.69 (10.68)	0.89 (17.44)	0.7031	0.05 (− 0.17, 0.27)
*School performance*					
Mother-adolescent dyad	69	18.30 (15.98)	0.54 (24.39)	0.8537	0.02 (− 0.19, 0.24)
Father-adolescent dyad	55	16.59 (17.85)	2.05 (24.39)	0.5365	0.08 (− 0.15, 0.32)
Mother–Father dyad	57	12.06 (13.56)	0.22 (18.22)	0.9279	0.01 (− 0.18, 0.21)
*Body image*					
Mother-adolescent dyad	75	18.83 (20.07)	− 7.50 (26.56)	0.0168	− 0.28 (− 0.49, − 0.08)
Father-adolescent dyad	63	26.39 (21.66)	− 4.96 (33.94)	0.2505	− 0.15 (− 0.44, 0.14)
Mother–father dyad	63	20.04 (18.99)	− 2.18 (27.64)	0.5331	− 0.08 (− 0.33, 0.17)

^a^Absolute difference =|Parent-proxy score—Adolescent score| or |Mother-proxy score—Father-proxy score|

^b^Directional difference = (Parent-proxy score—Adolescent score) or (Mother-proxy score—Father-proxy score)

^c^Effect size interpretation: negligible (|< 0.20|), small (|0.20–0.49|), moderate (|0.50–0.79|), and large (|≥ 0.80|)

**Fig. 1:**
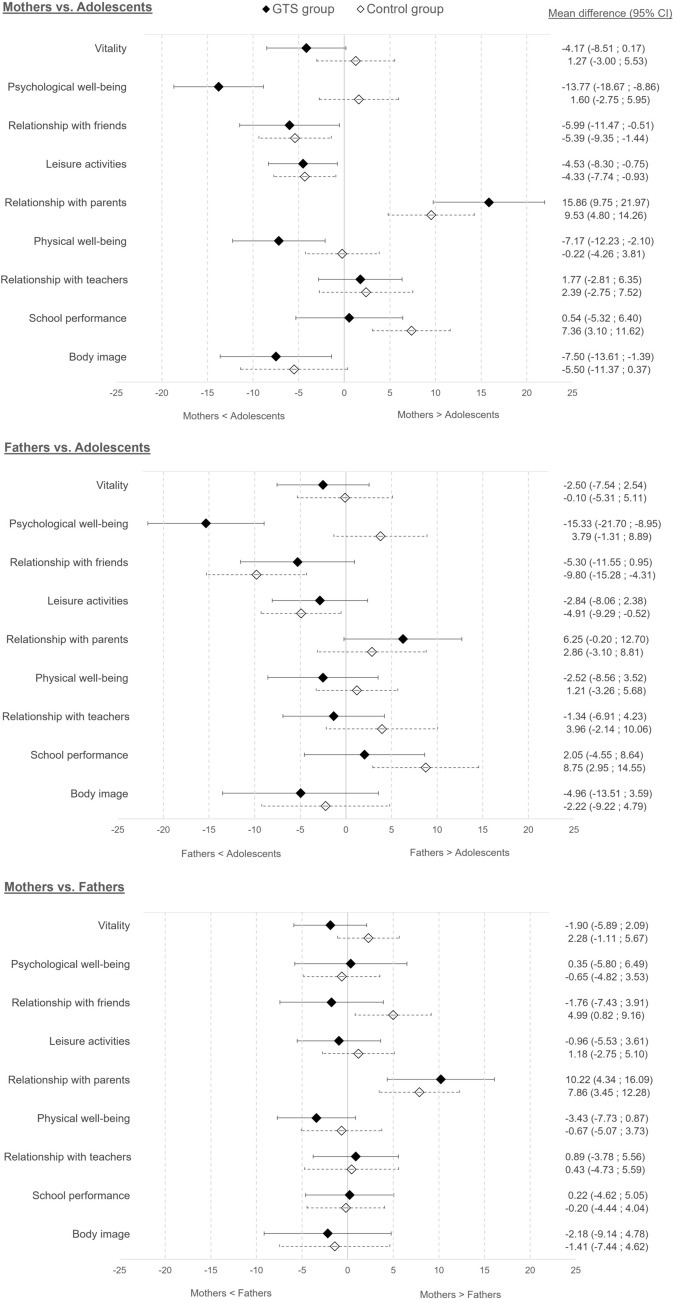
Mean direction differences adolescents’ HRQoL scores in mother-adolescent, father-adolescent and mother-father dyads in TS and control groups

Directional differences in adolescents’ HRQoL scores in mother-adolescent, father-adolescent and mother-father dyads of the TS group are presented in Table 3 and Fig. 1. Mothers’ proxy reports were significantly lower than adolescents’ self-reports, indicating an underestimation of adolescents’ HRQoL, for five of the nine scales: ‘Psychological well-being’ (p < 0.0001), ‘Relationship with friends’ (p = 0.0325), ‘Leisure activities’ (p = 0.0193), ‘Physical well-being’ (p = 0.0062) and ‘Body image’ (p = 0.0168). Effect size showed moderate underestimation for ‘Psychological well-being’ subscale (ES = − 0.65) and small underestimation for ‘Relationship with friends’ (ES = − 0.26), ‘Leisure activities’ (ES = − 0.28), ‘Physical well-being’ (ES = − 0.33) and ‘Body image’ subscales (ES = -0.28). Mothers significantly overestimated adolescents’ HRQoL only for ‘Relationship with parents’ subscale (p < 0.0001), with a moderate ES of 0.60. Fathers’ reports were significantly lower than adolescents’ self-reports only for ‘Psychological well-being’ subscale (p < 0.0001) with a moderate underestimation (ES = − 0.62). There was no significant difference between parents’ proxy-reports (mothers as fathers) and adolescents self-reports for ‘Vitality’, ‘Relationship with teachers’ and ‘School performance’ subscales. Regarding the parents dyad, mothers’ proxy reports were significantly higher than fathers’ ones for ‘Relationship with parents’ subscale (p = 0.0009) with a small overestimation (ES = 0.44), showing that mothers were more likely than fathers to overestimate the adolescent’s scores in this domain.

### Comparison of agreement and discrepancies between TS and control groups

The differences between the level of agreement in TS and control groups were non-significant for all HRQoL subscales (Table 2). However, some variations in ICC values have to be noted. Compared to the control group, agreement was better in the TS group between adolescents and mothers for ‘Vitality’, ‘Physical well-being' (moderate *vs.* fair agreement), ‘Relationship with teachers’ (good *vs.* moderate agreement), and ‘Body image’ (good *vs.* fair agreement) subscales. Agreement was better in father-adolescent dyads of the TS group for ‘Vitality’, ‘Relationship with friends’ and ‘Relationship with teachers’ subscales (moderate *vs.* fair agreement). Agreement in mother and father-adolescent dyads of the TS group was poorer for ‘Relationship with parents’ subscale (poor *vs.* fair agreement). Agreement between mothers and fathers was better in the TS group for ‘Relationship with friends’, ‘Relationship with teachers’ (good *vs.* moderate agreement), and ‘Physical well-being’ (good *vs.* fair agreement) subscales, and poorer for ‘Psychological well-being’ (moderate *vs.* good agreement) and ‘Relationship with parents’ (poor *vs.* moderate agreement) subscales.

In the mother-adolescent dyads, mean directional differences were significantly lower in the TS group for ‘Psychological well-being’ (p < 0.0001) and ‘Physical well-being’, (p = 0.0342) which indicated that TS mothers underestimated adolescents’ HRQoL while control mothers slightly overestimated adolescents’ HRQoL in psychological and physical domains (Fig. 1 and Supplementary Table 2). TS mothers seemed to better estimate adolescents’ HRQoL for ‘School performance’ than did control mothers, even if the difference was not statistically significant (p = 0.0627). In the father-adolescent dyads, the only significant difference was found for ‘Psychological well-being’ subscale (p < 0.0001): TS fathers underestimated adolescents’ HRQoL whereas control fathers overestimated it. No significant results was found in the mean directional differences for the mother-father dyads. However, TS parents tended to slightly underestimated the adolescents’ HRQoL for ‘Relationship with friends’ subscale while control parents overestimated it (p = 0.0573).

### Individual adolescents and parental factors related to dyads discrepancies in the TS group

The influence of factors on parent-adolescent and mother-father differences in scores were investigated for subscales where mean directional difference scores differed significantly between TS and control groups. TS mothers had significantly lower discrepancies for ‘Psychological well-being’ and ‘Physical well-being’ subscales and TS fathers had significantly lower discrepancies for ‘Psychological well-being’.

Larger mother-adolescent discrepancies for ‘Psychological well-being’ subscale (i.e. mother ratings lower than adolescent ratings) were associated with higher adolescent score in this subscale (standardized β = − 0.67, p < 0.0001), the presence of borderline or clinical ‘Internalizing symptoms’ on CBCL as rated by mothers (standardized β = − 0.33, p = 0.0009) and lower mother score in WHOQOL-BREF ‘Social relationships’ subscale (standardized β = 0.25, p = 0.0097). Adolescents’ gender and YGTSS ‘Phonic tics’ subscale were significantly associated with mother-adolescent discrepancies for ‘Psychological well-being’ subscale in bivariate analyses. Larger discrepancies were found in boys (p = 0.0208) and were associated with higher YGTSS ‘Phonic tics’ score (p = 0.0220). These factors related to adolescents were not selected in the final multivariate forward linear regression.

Larger mother-adolescent discrepancies for ‘Physical well-being’ subscale (i.e. mother ratings lower than adolescent ratings) were associated with higher adolescent score in this subscale (standardized β = − 0.60, p < 0.0001) and the presence of borderline or clinical ‘Internalizing symptoms’ on CBCL as rated by mothers (standardized β = − 0.37, p = 0.0007). Adolescents’ gender, YGTSS ‘Phonic tics’ and MOVES ‘Associated symptoms’ subscales were significantly associated with mother-adolescent discrepancies for ‘Physical well-being’ subscale in bivariate analyses. Larger discrepancies were found in boys (p = 0.0123), and were associated with higher YGTSS ‘Phonic tics’ score (p = 0.0390) as MOVES ‘Associated symptoms’ score (p = 0.0131). Nevertheless, these factors were not selected in the final multivariate forward model.

Larger father-adolescent discrepancies for ‘Psychological well-being’ subscale (i.e. father ratings lower than adolescent ratings) were only associated in the final multivariate model with higher adolescent score in this subscale (standardized β = − 0.65, p < 0.0001). In bivariate analyses, adolescents’ gender and time since first symptoms or diagnosis were significantly associated with father-adolescent discrepancies. Larger discrepancies were found in boys (p = 0.0003) and were associated with longer time since first symptoms (p = 0.0167) or diagnosis (p = 0.0293).

## Discussion

Regarding HRQoL in adolescents with TS, this is the first study to assess, in comparison to a healthy control group, agreement between self-, mother and father proxy-reports, agreement between mothers and fathers and also factors associated with higher discrepancies in TS dyads.

Interestingly, we showed that there was no difference in agreement between self-, mother and father proxy-reports in families of adolescents with TS compared to healthy control families. This suggests that parents of adolescents with TS are able to quite accurately perceive the difficulties these adolescents are encountering and to assess their adolescents’ quality of life.

In the TS families, the agreement between adolescents and mothers or fathers varied according to dimensions. Regarding ‘Leisure activities’ and ‘Relationship with teachers’ subscales, the agreement between parents’ proxy-reports (mothers as fathers) and adolescents self-reports was good. This could be explained because these are dimensions on which adolescents continue to "share" with parents or for which the evaluation can be based on more "objective" elements. This could also be explained by the fact that the parents of adolescents with TS would be even more involved in monitoring these aspects due to the health problems of these adolescents. On the contrary, agreement between adolescents and mothers or fathers, and between mothers and fathers was poor for ‘Relationship with parents’ subscale. Regarding the mother-adolescent dyad, mothers moderately overestimated adolescents’ HRQoL for this subscale. Regarding the parents dyad, mothers were more likely than fathers to overestimate the adolescent’s scores in this domain. Compared to the control group, agreement between all the dyads was poorer for ‘Relationship with parents’ subscale. This result is an advance compared to previous studies [5, 10–13]; indeed, the study of the 'Relationship with parents' subscale thanks to the VSP-A is particularly relevant in adolescence, a developmental stage during which relationships with parents change. This poor agreement can be partly linked to the developmental trajectory in adolescence and the evolution of the relationships with the parents at this stage of life, as described in general population [19]. However, the fact that the agreement was poorer in the TS group suggests that the ‘Relationship with parents’ dimension may also be partly impaired by TS including comorbid conditions.

The evaluation by the two parents is very interesting given the differences observed. If TS mothers had better concordance than fathers with adolescents, which was also the case among control families, mothers significantly underestimated quality of life of their adolescents in five of nine subscales (‘Psychological well-being’, ‘Relationship with friends’,’ Leisure activities’, ‘Physical well-being’ and ‘Body image’). By contrast, only ‘Psychological well-being’ subscale was underestimated by fathers.

Whereas control mothers and fathers slightly overestimated adolescents’ HRQoL in ‘Psychological well-being’ subscale as described in general population [19], TS mothers and fathers moderately underestimated it. Gün et *al*. [11] studying agreement between the child and adolescent with TS and ADHD and parent on HRQOL’s ratings concluded that psychosocial PedsQL score was higher in the child and adolescent ratings. Among 26 adolescents, Storch et *al*. indicated that “parents generally rated the adolescents’s QoL as being more negatively affected by their tic disorder than the youth endorsed”; note that Storch et *al*. did not distinguish whether the adolescent’s HRQoL was rated by the mother or the father and agreement was assessed using Pearson correlation coefficients and not intraclass correlation coefficients as in our study. [5]. On the other hand, the other studies did not identify the adolescent population or did not study agreement on this dimension [10, 12, 13].

We found in TS families that the mother ratings lower than adolescent ratings for ‘Psychological well-being’ and ‘Physical well-being’ subscales were associated to the presence of borderline or clinical ‘Internalizing symptoms’ on CBCL as rated by mothers. This could suggest that mothers linked those symptoms and adolescents’ HRQoL, which is consistent with the study of Storch et *al*. who reported a moderate relation between parent-rated internalizing symptoms and parents’ reports of child HRQOL in psychosocial, emotional and physical domains [5]. In addition, lower mothers self-reported HRQoL score in ‘Social relationships’ subscale of the WHOQOL-BREF were associated to larger mother-adolescent discrepancies for ‘Psychological well-being’ subscale.

We provided additional data by showing that mother and father ratings lower than TS adolescent ratings for ‘Psychological well-being’ subscale were associated with higher adolescent score in this subscale. This could be better understood by taking into account some specificities of adolescence. Many teenagers, becoming more independent, provide less information to their parents about their psychological state; parents therefore have less information to assess this dimension of adolescents' HRQoL and underestimated it.

Likewise, mother ratings lower than TS adolescent ratings were associated with higher adolescent score in ‘Physical well-being’ subscale. It seems that mothers in the TS group tended to worry more easily about their adolescents’ physical health. TS adolescents who evaluated this dimension as good were certainly more independent and communicated less with their mothers about their physical health. Thus mothers had less information provided by the adolescents to assess correctly this dimension and their concerns might lead to underestimate their adolescents’ HRQoL score in the ‘Physical well-being’ subscale.

When testing the effect of demographic and clinical factors on mother-adolescents agreement, the severity of phonic tics, assessing by the YGTSS, although significant in the bivariate analyses, was not selected in the multivariate models. This tic impairment scale is based on a single clinician hetero-evaluation. An assessment by the adolescents and the parents themselves would probably have been more appropriate. For example, the mini-Child Tourette Syndrome Impairment Scale could be used to assess tic-related and non-tic related impairment across school, home, and social domains [32].

### Strengths and limitations

We recruited a large sample of consecutive outpatients with TS aged 12–18 years and their parents and compared them with healthy adolescents matched for age, sex and family conditions. The method of recruiting adolescent healthy controls and their families was one of the strengths of our study since it smoothed the effects of age and sex on the HRQoL of adolescents and those of the place of residence and number of children on the parents [33]. Another strength of our study was the differentiation of the reports of mothers and fathers and the evaluation of the agreement between their reports, which no HRQoL study of adolescents with TS had done.

All the mothers and 84% of the fathers of the adolescents answered the questionnaires, which was an excellent rate compared to the literature where usually only one of the parents, often the mother, filled out the questionnaires [34–39].

The sample of adolescents with TS could be considered as closely representative because similar in terms of age at first symptoms and age at TS diagnosis to those described in clinical studies [40], and in terms of sex ratio and rates of co-occurring attention problems and OCD to those in population-based studies [41]. Compared to adolescents recruited exclusively in specialists clinics where more complex or severe cases are seen, our adolescents recruited from primary and secondary referral centers had lower mean YGTSS total score reflecting a mild to marked tic severity [42, 43]. Their medical treatment corresponds to the drugs usually prescribed in patients with TS or even with associated comorbidities [44, 45]. Finally, we used numerous analytical techniques at individual and group level to examine the agreement between self-, mother and father proxy-reports on HRQoL in adolescents with TS.

A limitation to this study is that adolescents’ HRQoL was not evaluated with a disease-specific quality of life instrument, like the GTS-QOL-French-Ado recently validated [46]. This questionnaire was published out after we had completed our recruitment, and no parent-proxy version was developed. Second, we could not exclude an under-representation of adolescents with mild symptoms although our sample of outpatients with TS was large and closely representative of the entire population of adolescents with TS.

Third, although none of control adolescents had tics, other diseases in control adolescents were not collected; so we could not ensure that they were all healthy. However, their medical treatments were collected and 18.7% of these 75 adolescents had at least one. In details, methylphenidate was taken by two adolescents, sodium valproate by one; treatments other than neuropsychiatric (i.e. mainly antihistaminic and bronchodilator) were taken by 12 adolescents. Two control adolescents were followed by a neurologist, three by a psychiatrist, and three by a psychologist. Therefore the sample of control adolescents could be considered as closely representative of adolescents in general population [47, 48].

Another limitation is that we have not corrected the p-values for multiple comparisons in statistical analysis. If making correction for multiple comparisons reduces the chance of making type I errors (that is the chance of incorrectly declaring a statistical significance), it increases the chance of making type II errors (that is the chance that effective differences are not discovered by statistical comparisons) [28, 29]. As our study was exploratory, we considered that the consequences of making a type I error were less important than making a type II error, and we not wanted to miss uncovering an effect worthy of further study [49].

## Conclusion

This study showed that there was no difference in agreement between self-, mother and father proxy-reports on adolescents’ HRQoL for families of adolescents with TS compared to healthy control families. However, TS mothers and fathers underestimated adolescents’ HRQoL in ‘Psychological well-being’ domain and TS mothers underestimated adolescents’ HRQoL in ‘Physical well-being ‘domain, while controls overestimated adolescents’ HRQoL in these subscales. Thus clinicians working with TS adolescents and their parents should take into account this point to provide comprehensive care and services. Regarding future studies, we draw attention that comprehensive evaluation of the various dimensions of adolescents’ HRQoL with TS requires the integration of the perspectives of both adolescents, mothers and fathers.

## Supplementary Information

Below is the link to the electronic supplementary material.

Supplementary file1 (DOCX 32 KB)

## Data Availability

The data supporting the findings of this study are available from the corresponding author upon reasonable request.
