# Supplementary material for: Agreement between self-, mother and father proxy-reports on health-related quality of life in adolescents with Tourette syndrome
Source: Eur Child Adolesc Psychiatry. 2024 Apr 13;33(11):3871–84. doi: 10.1007/s00787-024-02418-6 (PMC11588836; doi:10.1007/s00787-024-02418-6)
Supplement: Supplementary file 1 — Supplementary file1 (DOCX 32 KB) [file 787_2024_2418_MOESM1_ESM.docx]

**Agreement between self-, mother and father proxy-reports on health-related quality of life in adolescents with Tourette Syndrome**

Isabelle Jalenques, Candy Guiguet-Auclair, Dominique Morand, Fabien Bourlot, Sophie Lauron, Nathan Mitelman, Andreas Hartmann, Fabien Rondepierre, The Syndrome de Gilles de La Tourette Study Group

**Table of Contents**

Supplementary Table 1: Adolescents self-reports, mothers and fathers proxy-reports of adolescents’ HRQoL

Supplementary Table 2: Comparison between TS and control group of the directional differences of adolescents’ HRQoL scores in mother-father, father-adolescent and mother-father dyads

**Supplementary Table 1**: Adolescents self-reports, mothers and fathers proxy-reports of adolescents’ HRQoL

|  | Adolescents | | Mothers | | Fathers | |
| --- | --- | --- | --- | --- | --- | --- |
| VSP-A / VSP-P scales | n | Mean (SD) | n | Mean (SD) | n | Mean (SD) |
| Vitality |  |  |  |  |  |  |
| TS group | 75 | 60.3 (21.0) | 75 | 56.2 (19.9) | 62 | 58.4 (17.1) |
| Control group | 75 | 72.7 (16.7) | 75 | 73.9 (13.2) | 62 | 72.7 (15.7) |
| Psychological well-being |  |  |  |  |  |  |
| TS group | 75 | 68.0 (24.6) | 74 | 54.5 (23.4) | 61 | 52.9 (20.3) |
| Control group | 75 | 73.3 (16.2) | 75 | 74.9 (18.1) | 62 | 76.7 (18.0) |
| Relationship with friends |  |  |  |  |  |  |
| TS group | 75 | 56.4 (22.9) | 71 | 50.5 (24.8) | 61 | 50.2 (24.3) |
| Control group | 75 | 74.8 (17.6) | 75 | 69.4 (15.6) | 61 | 66.3 (17.3) |
| Leisure activities |  |  |  |  |  |  |
| TS group | 75 | 52.2 (23.0) | 75 | 47.6 (25.6) | 63 | 48.8 (26.0) |
| Control group | 75 | 64.3 (19.4) | 75 | 60.0 (21.5) | 62 | 59.9 (22.0) |
| Relationship with parents |  |  |  |  |  |  |
| TS group | 75 | 52.3 (22.3) | 75 | 68.1 (16.7) | 62 | 59.5 (16.7) |
| Control group | 75 | 56.6 (20.2) | 75 | 66.2 (15.0) | 62 | 59.0 (19.1) |
| Physical well-being |  |  |  |  |  |  |
| TS group | 75 | 67.3 (21.9) | 75 | 60.2 (20.5) | 62 | 63.8 (18.1) |
| Control group | 75 | 74.3 (14.0) | 75 | 74.1 (14.8) | 62 | 76.3 (15.7) |
| Relationship with teachers |  |  |  |  |  |  |
| TS group | 67 | 60.0 (22.4) | 70 | 62.1 (22.2) | 57 | 58.8 (19.6) |
| Control group | 74 | 57.9 (22.5) | 69 | 59.7 (17.9) | 61 | 60.9 (18.1) |
| School performance |  |  |  |  |  |  |
| TS group | 70 | 56.6 (28.3) | 71 | 57.0 (24.1) | 58 | 56.9 (23.1) |
| Control group | 73 | 62.8 (23.5) | 75 | 70.0 (19.5) | 62 | 70.8 (18.4) |
| Body image |  |  |  |  |  |  |
| TS group | 75 | 73.2 (31.2) | 75 | 65.7 (29.4) | 63 | 67.9 (26.9) |
| Control group | 75 | 82.3 (20.9) | 75 | 76.8 (22.9) | 62 | 80.2 (24.7) |

**Supplementary Table 2**: Comparison between TS and control group of the directional differences of adolescents’ HRQoL scores in mother-father, father-adolescent and mother-father dyads

|  | Mother-Adolescent dyad | | | Father-Adolescent dyad | | | Mother-Father dyad | | |
| --- | --- | --- | --- | --- | --- | --- | --- | --- | --- |
|  | TS group | Control group |  | TS group | Control group |  | TS group | Control group |  |
| VSP-A / VSP-P scales | Mean (SD) | Mean (SD) | *p-value* | Mean (SD) | Mean (SD) | *p-value* | Mean (SD) | Mean (SD) | *p-value* |
| Vitality | -4.17 (18.86) | 1.27 (18.55) | *0.0773* | -2.50 (19.85) | -0.10 (20.52) | *0.5094* | -1.90 (15.71) | 2.28 (13.35) | *0.1135* |
| Psychological well-being | -13.77 (21.17) | 1.60 (18.91) | *<0.0001* | -15.33 (24.90) | 3.79 (20.08) | *<0.0001* | 0.35 (23.99) | -0.65 (16.43) | *0.7896* |
| Relationship with friends | -5.99 (23.15) | -5.39 (17.20) | *0.8604* | -5.30 (24.40) | -9.80 (21.43) | *0.2819* | -1.76 (21.55) | 4.99 (16.28) | *0.0573* |
| Leisure activities | -4.53 (16.40) | -4.33 (14.80) | *0.9393* | -2.84 (20.73) | -4.91 (17.27) | *0.5471* | -0.96 (18.16) | 1.18 (15.46) | *0.4808* |
| Relationship with parents | 15.86 (26.55) | 9.53 (20.56) | *0.1047* | 6.25 (25.41) | 2.86 (23.46) | *0.4411* | 10.22 (23.12) | 7.86 (17.38) | *0.5233* |
| Physical well-being | -7.17 (22.01) | -0.22 (17.54) | *0.0342* | -2.52 (23.78) | 1.21 (17.60) | *0.3230* | -3.43 (16.93) | -0.67 (17.33) | *0.3722* |
| Relationship with teachers | 1.77 (18.63) | 2.39 (21.21) | *0.8573* | -1.34 (20.21) | 3.96 (23.61) | *0.2061* | 0.89 (17.44) | 0.43 (19.63) | *0.8947* |
| School performance | 0.54 (24.39) | 7.36 (18.26) | *0.0627* | 2.05 (24.39) | 8.75 (22.47) | *0.1277* | 0.22 (18.22) | -0.20 (16.71) | *0.8956* |
| Body image | -7.50 (26.56) | -5.50 (25.52) | *0.6389* | -4.96 (33.94) | -2.22 (27.58) | *0.6213* | -2.18 (27.64) | -1.41 (23.75) | *0.8675* |

Directional Difference = (Parent-proxy score - Adolescent score) or (Mother-proxy score - Father-proxy score)
